# Advantages and Disadvantages of Inclisiran: A Small Interfering Ribonucleic Acid Molecule Targeting PCSK9—A Narrative Review

**DOI:** 10.1155/2022/8129513

**Published:** 2022-02-10

**Authors:** Iveta Merćep, Nikolina Friščić, Dominik Strikić, Željko Reiner

**Affiliations:** ^1^Department of Clinical Pharmacology, University Hospital Centre Zagreb, Croatia; ^2^Department of Internal Medicine, University of Zagreb, School of Medicine, Croatia; ^3^Resident of the Transfusion Medicine, University Hospital Centre Zagreb, Croatia; ^4^Resident of the Clinical Pharmacology, University Hospital Centre Zagreb, Croatia; ^5^Department of Metabolism, University Hospital Centre Zagreb, Croatia

## Abstract

As dyslipidemias remain one of the main risk factors for developing cardiovascular disease, the question of maintaining optimal lipid levels with pharmacotherapy remains a subject of interest worldwide. In contrast to conventional pharmacotherapy, human monoclonal antibodies directed against proprotein convertase subtilisin/kexin type 9 (PSCK9) and small interfering RNA- (siRNA-) based drug targeting PCSK9 represent a new strategy for managing lipid disorders and reducing cardiovascular risk. Inclisiran is a long-acting, synthetic siRNA that targets hepatic production of PCSK9 and consequently causes a reduction in LDL-C concentrations by approximately 50% compared to placebo. The structural modification of inclisiran has led to better stability and prolonged biological activity of the drug. The main advantage over conventional pharmacotherapy and anti-PCSK9 monoclonal antibodies is its favorable administration regimen (0–90–180 days), which should lead to much better compliance. Clinical trials conducted so far have confirmed the tolerability and efficacy of inclisiran in long-term PCSK9 and LDL-C level reductions. Moreover, a short-term follow-up on the safety of inclisiran showed a relatively good safety profile of the drug. However, it is still of great importance for ongoing and forthcoming clinical trials to be continued on a larger group of patients in order to assess long-term tolerability, efficacy, and safety of inclisiran.

## 1. Introduction

Dyslipidemia is one of the most important risk factors for cardiovascular disease (CVD) [1]. The role of low-density lipoprotein cholesterol (LDL-C) in the development of atherosclerosis is well known to be the benefit of lowering elevated LDL-C levels in the reduction of cardiovascular (CV) risk [2]. The current European Society of Cardiology/European Society of Atherosclerosis guidelines for the management of dyslipidemia [3] emphasize on adequate control and treatment of dyslipidemia, particularly on LDL-C lowering. The most recent relevant position papers also emphasize the importance of LDL-C lowering, particularly in high, very high, and extremely high-risk patients [4]. Pharmacotherapy with statins is still the gold standard in the treatment of hypercholesterolemia and particularly elevated LDL-C; however, available data from surveys, prospective studies, and clinical registries provide insight that some patients do not reach their therapeutic goals even with the highest tolerated doses of statins [5–7]. Moreover, chronic statin therapy is associated with adverse effects, such as myalgia and myopathies, and its occurrence is one of the major reasons for discontinuation of the therapy [8, 9]. The limitations of statin therapy have led to the discovery of now established agents that target proprotein convertase subtilisin/kexin type 9 (PCSK9) [10]. PCSK9 is a protein involved in the regulation of LDL-C metabolism by interfering with LDL receptors, consequently causing a reduction in plasma LDL-C levels and an increase in circulating LDL-C levels [11]. Therefore, inhibition of PCSK9 has been proposed as a new therapeutic approach for treating lipid disorders. So far, only therapy with monoclonal antibodies, alirocumab and evolocumab, has been approved for treating patients with hypercholesterolemia. The frequent administration and possible lack of adherence during long-term treatment with monoclonal antibodies have led to the development of new agents targeting PCSK9, such as inclisiran—siRNA. Inclisiran is a new promising agent, synthetic siRNA, currently being evaluated in phase III clinical trials. Its mechanism of action reduces intra- and extracellular PCSK9 levels unlike monoclonal antibodies that reduce only extracellular PCSK9 levels [12]. This specific mechanism of action causes considerable and long-lasting reduction in LDL-C concentrations while on inclisiran [11].

## 2. Methods

This narrative review was performed by searching PubMed, Scopus, Embase, and Web of Science from inception to May 1, 2021, using the following keywords in titles and abstracts: “inclisiran” AND “ALN-PCSsc.”

### 2.1. Inclisiran: A siRNA Molecule

Inclisiran is a long-acting, short-chain siRNA directed against PCSK9 protein. siRNA molecules use the natural pathway of selective gene expression silencing. Inclisiran inhibits the expression of PCSK9 by binding specifically to the mRNA precursor of PCSK9 protein and causing its degradation. This specific siRNA molecule consists of two complementary ribonucleic acid strands: a guide strand and a passenger strand. When incorporated into hepatocytes, the guide strand binds to the RNA-induced RISC. Consequently, hybridization with the complementary mRNA for PCSK9 induces its degradation. Degradation of PCSK9 mRNA reduces the synthesis and secretion of PCSK9 by limiting its translation. It was noticed that the silencing complex remained active even after mRNA degradation, leading to the conclusion that this may be the reason for the long-term efficacy of inclisiran. Due to its specific mechanism of action, inclisiran causes a reduction in both intracellular and extracellular PCSK9 protein levels, resulting in a considerable and long-lasting reduction in plasma LDL-C concentrations [11–13]. This mechanism might be its advantage, but we still have to wait for the trial results of the long-term effects for the inclisiran treatment [14].

### 2.2. Pharmacodynamics of Inclisiran

Unlike the other siRNA such as ALN-PCS molecule, inclisiran has an additional synthetic triantennary carbohydrate, N-acetylgalactosamine (GalNAc) [11, 12]. This carbohydrate is complementary to asialoglycoprotein receptors on hepatocytes, causing inclisiran uptake directly and specifically into hepatocytes. Additionally, the presence of GalNAc significantly increases the clinical and long-term efficacy of inclisiran by increasing adhesion to the hepatocytic cellular membrane [11]. Studies have shown that no drug molecules were found in the serum 24 h after intravenous drug administration, suggesting a conclusion of specific drug influx into the liver [15]. This specificity to hepatocytes could decrease the risk of possible adverse effects on other organs. In preclinical studies on animal models, a linear relationship between dose and efficacy was observed. To increase the stability of the drug, molecules in the polynucleotide strand were substituted with 2′-O-methylnucleotides or 2′-O-fluoronucleotides. The therapeutic dose of 1 mg/kg of body mass was associated with 50% PCSK9 inhibition, and the highest administered dose of 3 mg/kg of body mass showed an inhibition of PCSK9 of 85% and a reduction in LDL-C concentrations of 60% [16]. It is also important to mention that the spherical structure of the precursor of inclisiran inserted into the lipid nanoparticle, the ALN-PCS molecule, caused a reduction of 70% in both PCSK9 mRNA and protein concentrations and a 60% reduction in LDL-C concentrations in animal models with a duration of three weeks [16]. Studies on healthy volunteers showed a 70% reduction in PCSK9 protein after 60 minutes of drug infusion and a 40% reduction in LDL-C levels on the third day of follow-up [17]. Inclisiran has an additional clinical benefit compared to its precursor, ALN-PCS. The modified structure of inclisiran allows subcutaneous administration of the drug, unlike ALN-PCS, which requires intravenous administration [11].

### 2.3. Pharmacokinetics of Inclisiran

The highest concentration of ALN-PCS is achieved after termination of a 60-minute intravenous drug administration [11]. The maximal concentration and area under the curve of drug concentration correlate relatively linearly with the drug dose [17]. Preclinical studies on animal models showed that the largest reduction in PCSK9 and LDL-C concentrations was observed approximately 20 days after subcutaneous drug administration. The reduction was not dependent on the administered drug dose. Studies have also shown that the duration of a lipid-lowering effect correlates with drug dose: the higher the dose of ALN-PCSSC, the longer the effect. A small but progressive increase in LDL-C concentration was also observed 90 to 120 days after drug administration [18]. Unlike ALN-PCS, modification of the molecular structure of ALN-PCSsc enabled better stability and prolonged biological activity of the drug [19]. Because of its favorable pharmacokinetics, inclisiran requires subcutaneous administration at 0 and three months and then every six months reducing LDL-C by approximately 50% in patients with high and very high cardiovascular risk or with a diagnosis of familial hypercholesterolemia as well as in patients intolerant to statins.

### 2.4. Clinical Trials with Inclisiran

Preclinical studies and phase 1 and phase 2 clinical trials have been performed to date. Phase 3 clinical trials are ongoing. Preclinical studies showed a linear correlation between the dose of the drug and its efficacy, which initiated clinical trials to obtain regulatory approval for the use of inclisiran in hypercholesterolemia therapy [11]. A phase 1 trial (NCT01437059) was performed on 32 healthy volunteers with LDL-C concentrations over 116 mg/dl. They received a single intravenous infusion of different doses of inclisiran. The primary endpoint was the analysis of drug tolerance and safety, and the secondary endpoint was the determination of pharmacodynamics and pharmacokinetics of the drug. Administration of the highest dose of inclisiran (0.4 mg/kg) caused a reduction in serum PCSK9 by approximately 70% and LDL-C by 40%. The lipid-lowering effect was greatest when the baseline LDL-C concentration was the highest [17]. Another phase 1 trial on the safety of subcutaneous treatment with inclisiran was performed on 24 healthy volunteers with LDL-C levels higher than 100 mg/dl (2.6 mmol/l). They were randomly assigned to receive single-dose injections of inclisiran (25, 100, 300, 500, and 800 mg) or multiple-dose injections (125 mg once a week for four doses, 250 mg twice a week for two doses, and 300 mg or 500 mg monthly for two doses) [13, 18]. The study showed that a single dose of 100 mg of inclisiran or higher was necessary to significantly reduce LDL-C levels. In addition, a significant reduction in PCSK9 expression was observed after a single dose of 300 mg of inclisiran. Furthermore, the maximal reduction of PCSK9 was 74.5% after a 300 mg dose of inclisiran at day 84, and the maximal reduction of LDL-C levels was 50.6% after a 500 mg dose of inclisiran. The administration of a single dose of 300 mg inclisiran or higher was able to maintain reduced levels of PCSK9 and LDL-C for more than 180 days. Compared to the multiple-dose subgroup, the highest LDL-C reduction was observed in the group receiving a 300 mg dose of inclisiran and 2 doses per month. The reduction in LDL-C was 83.8% and 59.7%, respectively, at day 84, and the reduction persisted for 196 days after administration of the first dose [11, 18]. The first phase 2 trial on the lipid-lowering effect of inclisiran was ORION-1 (NCT02597127). This multicenter, randomized, placebo-controlled study confirmed the findings of the preclinical studies. It was performed on 501 patients at high risk of CVD with elevated baseline serum LDL-C concentration despite the maximal tolerated dose of conventional lipid-lowering therapy. The LDL-C concentration threshold in patients with CVD was >70 mg/dl, and in patients without CVD, it was >100 mg/dl [11, 13, 20]. Patients were randomly assigned to groups receiving a single dose of placebo or inclisiran (200, 300, or 500 mg) or two doses (1st and 90th day) of placebo or inclisiran (100, 200, or 300 mg). The change in LDL-C concentration on day 180 was the primary endpoint of this trial.

After 30 days of the first inclisiran administration, the concentration of PCSK9 was reduced by 66.2%-74.0% and the concentration of LDL-C was reduced by 44.5%-50.5% depending on the administered dose. Major LDL-C reduction was observed after a double dose of 300 mg of inclisiran. On day 180, in the group of patients who received the aforementioned dose, the mean reduction in PCSK9 was 69.1% and the reduction in LDL-C was 52.6%. Furthermore, during a 240-day follow-up period, levels of LDL-C were dose-dependent and varied between 26.7% and 47.2%. However, significant reductions in PCSK9 and LDL-C concentrations remained persistent in all dose subgroups. Compared to other anti-PCSK9 monoclonal antibodies, the percentage of LDL-C and PCSK9 concentration reduction was similar, but the effect of inclisiran lasted significantly longer. Data derived from this trial suggested that administration of inclisiran every six months may be sufficient [11, 21]. In an article in the *New England Journal of Medicine*, in April 2020, derived data from two phase 3 trials of inclisiran in patients with elevated LDL-C levels were published. These trials were placebo-controlled, double-blind, and randomized. The ORION-10 trial (NCT03399370) included 1561 patients in North America, and the ORION-11 trial (NCT03400800) included 1617 patients worldwide (non-USA). In the ORION-10 trial, patients with atherosclerotic cardiovascular disease (ASCVD) were enrolled, and the ORION-11 trial included patients with atherosclerotic cardiovascular disease (ASCVD) or atherosclerotic cardiovascular disease risk (ASCVD risk) equivalent to elevated LDL-C levels despite treatment with maximal doses of statins. The endpoint of these trials was to estimate the efficiency, safety, and tolerability of treatment with inclisiran. The placebo-corrected percentage change in LDL-C levels from baseline to day 510 and the time-adjusted percentage change in LDL-C levels from baseline between day 90 and day 540 were included as coprimary trial endpoints [13, 22]. In these trials, patients were randomly assigned to either the inclisiran or placebo groups [3]. Subcutaneous injection of 284 mg of inclisiran (equivalent 300 mg inclisiran sodium) or placebo was administered on day 1, day 90, and every 6 months thereafter for a period of 540 days. The results showed that on day 510, the reduction in LDL-C levels with inclisiran was 52.3% in the ORION-10 trial and 49.9% in the ORION-11 trial. Moreover, the occurrence of adverse effects was generally similar in the inclisiran and placebo groups in both trials. However, injection-site adverse effects were more frequent in the inclisiran group (2.6% vs. 0.9% in the ORION-10 trial and 4.7% vs. 0.5% in the ORION-11 trial) [22]. It is also important to mention that there are several more phase 3 clinical trials with inclisiran, two of them in familial hypercholesterolemia (FH) patients. ORION-9 is a trial that included about 482 patients with heterozygous FH in North America, Israel, Europe, and South Africa, and ORION-5 is a trial with 60 patients with homozygous FH in North America, Europe, and the Middle East [13]. Their primary endpoint was the percentage change in LDL-C levels at day 510 and time-adjusted percentage change between day 90 and day 540 (ORION-9), as well as an analysis of LDL-C change during the treatment (ORION-5) [23]. A recent study has summarized the results of ORION-10, ORION-11, and ORION-9 trials, confirming that inclisiran decreased LDL-C by 51% compared to the placebo, but that it also decreased total cholesterol (37%), apolipoprotein B (41%), and non-HDL-C (45%), which were associated with a 24% decrease in cardiovascular events, such as cardiac death, cardiac arrest, myocardial infarction, or stroke [24].

### 2.5. Adverse Effects of Inclisiran

Compared to conventional oral pharmacotherapy, injectable drugs are generally more frequently associated with an increased risk of adverse effects [25]. However, adverse effects of inclisiran are so infrequent that they require long clinical trials with large sample sizes to detect. Among the most frequent adverse effects noted in studies with inclisiran were local reactions such as mild self-limiting rash and hyperpigmentation, musculoskeletal pain, headache, cough, and back pain along with acute nasopharyngitis or hiccups [17, 18, 21]. In currently published studies on inclisiran, adverse effects occurred with similar frequencies in both placebo- and drug-treated arms [11]. In the ORION-1 trial, the overall occurrence of adverse effects was 76% in both placebo and inclisiran arms, but severe adverse effects were observed in only 8% in the placebo group and 11% in the inclisiran group of patients [21, 26]. Local reactions at the injection site occurred in 3% of the patients treated with the drug. However, the overall incidence of adverse effects was not significantly different between the drug-treated and placebo groups. Only one patient experienced an asymptomatic increase in *γ*-glutamyltransferase and alanine aminotransferase levels, which was associated with parallel treatment with atorvastatin rather than inclisiran itself [18]. It is important to mention that none of the patients involved in the studies with inclisiran discontinued therapy because of adverse effects. Nevertheless, there are a few more adverse effects that should be mentioned when considering the administration of inclisiran. So far, no symptoms of immune system activation have been observed, and there is no possible prothrombotic activity after RNA-based therapy. The possibility of prothrombotic activity may be connected to phosphorothioate modifications of RNA by triggering activation of platelet-activating factors leading to thrombosis despite the therapeutic range of drug concentrations [27]. Furthermore, the development of siRNA-induced peripheral neuropathy, which was observed after administration of siRNA, was not observed in any of the trials with inclisiran [11]. Inclisiran has also been proven to be safe in patients with renal function impairment, and no dose adjustment for these patients is needed [28].

## 3. Discussion

Based on data from all three phase III ORION studies, that is ORION-9, ORION-10, and ORION-11, which have confirmed the robust LDL-C lowering with inclisiran (284 mg) in the long-term with reduced plasma PCSK9 levels by approximately 80%, it seems that inclisiran is the first-in-class siRNA-based drug, which is not only efficient in changing the lipoprotein profile into a favorable one in patients with dyslipidemia, particularly in those with elevated LDL-C, but also has a very good safety profile [23, 29]. Therefore, inclisiran has been approved by the European Medicines Agency for use in the European Union in adults with primary hypercholesterolemia (heterozygous familial and nonfamilial) or mixed dyslipidemia as an adjunct to diet on December 9, 2020, and the drug is entering European markets [30]. In patients who are statin-intolerant or for whom statins are contraindicated, inclisiran can be used alone or in combination with other lipid-lowering drugs [31–33]. Although a new drug application (NDA) for inclisiran in patients with atherosclerotic CVD and familial hypercholesterolemia was submitted to the United States Food and Drug Administration in December 2019, the approval process has been delayed because of COVID-19-related travel restrictions and further inspection delays in manufacturing [34]. Several questions remain unanswered. One is whether inclisiran can decrease the risk of cardiovascular events; that is, whether this drug has a beneficial effect on hard endpoints and whether inclisiran can decrease cardiovascular and total mortality. These answers are expected from ongoing multinational ORION-4 and ORION-5 trials that assess the impact on cardiovascular outcomes in adults with established atherosclerotic CVD and in adults with homozygous familial hypercholesterolemia, respectively [30]. Another important question is whether inclisiran is safe enough to be used in younger patients with primary hypercholesterolemia. Therefore, placebo-controlled, multinational, phase III ORION-13 (NCT04659863) and ORION-16 (NCT04652726) trials, which are only recruiting patients, will assess short-term efficacy of inclisiran in adolescents aged 12 to 17 years with homozygous and heterozygous familial hypercholesterolemia, respectively, and elevated LDL-C on stable standard of care lipid-lowering therapy. If these issues are not resolved by the ongoing trials, this might be to the disadvantage of inclisiran. A disadvantage might also be exactly like this, which is considered to be a big advantage of inclisiran: a long-lasting effect requiring a stable therapy by injecting the drug only once in three to six months. The same extended duration of action that is a potential benefit of inclisiran may with respect to efficacy be detrimental because of possible prolonged adverse effects, which may be challenging to reverse. In such a case, the activity of inclisiran persists for six months without any possibility of intervening and stopping the possible adverse effects.

## 4. Conclusion

Inclisiran is a long-acting siRNA targeting PCSK9 that is administered by subcutaneous injections in quite favorable dosing regimens (0–90–180 days) using *N*-acetylgalactosamine (GalNAc) delivery technology. This drug decreases the expression of PCSK9 mRNA by RNAi, reducing the degradation of LDL receptors and increasing the uptake of LDL particles in hepatocytes, thus lowering elevated plasma LDL-C levels. Inclisiran robustly reduced LDL-C levels by approximately 50% compared to placebo. Although it is slightly lower than monoclonal antibody therapy, the advantage of treatment with inclisiran is that, in stable treatment, it is administered subcutaneously only once every three to six months, which assures improved compliance compared to daily oral agents in the short term and possibly over the long term. Disadvantages of inclisiran are possible adverse effects that might be noticed in the future after several years of treatment because the activity of inclisiran persists for six months making it challenging to reverse any potential adverse effects of long duration. Furthermore, in all trials so far, inclisiran was used together with oral agents, so in practice, at least initially inclisiran would be used together with oral agents, and not as a replacement treatment. However, as already mentioned, data on hard outcomes for inclisiran treatment are still awaited.
